# Association of surgeon volume with complications following direct anterior approach (DAA) total hip arthroplasty: a population-based study

**DOI:** 10.2340/17453674.2024.41506

**Published:** 2024-09-10

**Authors:** Pakpoom RUANGSOMBOON, Elmunzar BAGOURI, Daniel PINCUS, J Michael PATERSON, Bheeshma RAVI

**Affiliations:** 1 Sunnybrook Health Sciences Centre, Division of Orthopaedic Surgery, University of Toronto, Toronto, Ontario, Canada; 2 Department of Orthopaedics Surgery, Siriraj Hospital, Mahidol University, Thailand; 3 ICES, Toronto, Canada

## Abstract

**Background and purpose:**

Total hip arthroplasty (THA) can be performed through various surgical approaches, including direct anterior (DAA). DAA-THA may offer faster recovery but carries a higher risk of complications, which may be mitigated by surgeon volume and experience. We examined the association of surgeons’ annual surgical volume with major complications after DAA-THA in a population-based sample.

**Methods:**

A population-based retrospective cohort study was carried out on primary DAA-THA patients in Ontario between April 2016 and March 2021. We used restricted cubic splines to visually define the association between annual DAA surgeon volume and the risk of major surgical complications (fractures, dislocations, infections, and revisions) within 1 year of surgery. We further compared the complication rates amongst different DAA volume categories (< 30, 30–60, and > 60 cases/year).

**Results:**

The study encompassed 9,672 DAA-THA patients (52% female, median age 67 years). We showed a sharp decline in the probability of complications as the surgical volume of DAA-THA increased within the lower range of 0–30 cases/year; the probability slightly increased after the surgical volume exceeded 60 cases/year. The overall complication rates were 3.09%, 2.24%, and 2.18% for the surgical experience group of < 30 cases/year, 30–60 cases/year, and > 60 cases/year, respectively.

**Conclusion:**

There was an inverse relationship between surgical volume and complication rates in DAA-THA within the lower volume ranges. Maintaining a surgical volume of at least 30 DAA-THA cases/year can minimize complications, emphasizing the importance of surgical volume in this approach.

Various surgical approaches exist for total hip arthroplasty (THA), each presenting benefits and risks [1]. A surging trend in THA is the emphasis on sparing muscle dissection during surgery. Techniques such as the “sparing piriformis and internus, repair externus” (SPAIRE) for the posterior approach [2,3] and the direct anterior approach THA (DAA-THA) have become increasingly popular. Unlike traditional lateral or classic posterior approaches that often necessitate some muscle dissection, these methods utilize intermuscular techniques to minimize trauma around the hip [2,3]. Consequently, DAA-THA may contribute to a more rapid recovery and rehabilitation in the immediate to early postoperative phase [4]. However, 78% of hip surgeons expressed their concerns on utilizing DAA-THA for its lack of evidence on superior efficacy over alternative techniques and the fear of increased complications [5]. A large prior population-based study examined the outcomes of 30,098 adult patients and found that patients who underwent DAA-THA faced a 2–3 times higher risk of major complications compared with those treated with muscle-splitting approaches [6].

While the advantages of DAA-THA may be appealing, achieving surgical expertise on this technique involves significant challenges, particularly in terms of the learning curve and the precision of the surgical technique [7-9]. To the best of our knowledge, there has been no study that explicitly examines how a surgeon’s expertise or experience affects the results or complications of DAA-THA across a large and diverse population-based patient group and varied surgeon population. Therefore, we aimed to assess the association between the surgeon’s experience, represented by the annual surgical volume for DAA-THA, and the occurrence of postoperative major complications.

## Methods

### Data sources and setting

This was a population-based retrospective cohort study of patients undergoing primary DAA-THA in Ontario (Canada’s most populous province) from April 1, 2016, to March 30, 2021. All DAA-THAs were executed within Ontario’s single-payer healthcare system. Data was sourced from ICES (www.ices.on.ca), a non-profit research institute endorsed by the Ontario Ministry of Health. This database encapsulates Ontario residents’ medical, physician, hospital, and demographic information. In these databases, we used validated algorithms that were previously employed to identify patients undergoing DAA-THA, covariates, and the study outcomes [6,10].

This study was conducted according to the STROBE guidelines for observational studies [11].

### Patients

The study included participants aged 18 years and older from Ontario who were diagnosed with primary hip osteoarthritis (OA) and were undergoing elective primary DAA-THA. Observations for complications were carried out for 1 year, ending on March 31, 2022.

### Main exposure

We used the surgeon’s annual surgical volume as the determinant of their experience. We counted both primary and revision DAA-THA performed by the primary surgeon in the 365 days immediately preceding the index procedure and defined this number as the surgeon volume. Based on the restricted cubic spline, surgeon volume was subsequently categorized into 3 groups for analysis: < 30, 30–60, and > 60 cases/year.

### Outcomes

The primary study outcome was major surgical complications within 1 year following DAA-THA. A composite of 4 major complications was predetermined: revision arthroplasty, fracture, dislocation, and deep surgical infections, which were selected due to their significant impact on patient recovery, prognosis, and healthcare resource utilization. The 4 major complications were identified via the ICES database. “Revision arthroplasty” includes any revision surgery involving the acetabular component, replacement of the acetabular liner, revision of the femoral stem, or a complete revision of all components. “Fracture” refers specifically to periprosthetic fractures and does not include spinal osteoporotic fractures or late pelvic fractures. “Dislocation” captures instances of hip dislocation following the index procedure. “Deep surgical infections” includes any deep surgical site infections and periprosthetic joint infection identified postoperatively. Secondary outcomes included return to the emergency department (ED) within 30 days of surgery, readmission within 30 days, and mortality within 1 year. Outcomes included overall cases and were also stratified by surgeon volume groups.

### Covariates

Using validated algorithms, patients with a history of cardiovascular diseases, congestive heart failure, diabetes, hypertension, and chronic obstructive pulmonary disease were identified. Additional comorbidities from hospital discharge summaries 3 years prior to the DAA-THA were measured using the Charlson Comorbidity Index (CCI) [12]. Patients were categorized as “frail” using the Johns Hopkins ACG System (Version 10) [13]. The “Neighborhood Income Quintile” served as a socioeconomic status indicator [14]. Teaching hospitals, institutions training medical residents and fellows, were also identified.

### Statistics

Baseline attributes of the cohort were reported using descriptive statistics by surgeon volume group. We employed multivariate regression analyses incorporating the “picks the spots” macro algorithm with restricted cubic splines with 4 knots to estimate the predicted probability of complications and determine its association with surgeon volume [15]. This spline was used to inform the categorization of the cohort by surgeon volume; after stratification, we employed generalized estimating equations (GEE) to compare outcomes between groups before and after controlling for relevant confounders (patient age, sex, comorbidity, teaching hospital status) and for clustering by primary surgeon. The lowest surgeon volume group (< 30 cases/year) was used as the reference group a priori.

Crude and adjusted complication rates were compared across surgeon volume categories. Additionally, we examined the effect of teaching hospital status on outcomes through an unadjusted analysis. This analysis sought to determine whether the surgical setting influences complication rates, thereby providing a more comprehensive understanding of the variables impacting patient outcomes.

All analyses were performed at ICES using SAS version 9.3 (SAS Institute, Cary, NC, USA). The 2-sided type I error probability was set at 0.05. Missing data, which was less than 1% for all variables considered, was excluded from the regression models.

### Ethics, registration, data sharing, funding, use of AI, and disclosures

Use of the data in this project was authorized under section 45 of Ontario’s Personal Health Information Protection Act, which does not require review by a Research Ethics Board or individual patient consent. This study was an unfunded study. There is no patient-level data sharing for this research. Two authors of this study (DP, BR) were granted complete access to all the data in the study and assume accountability for the data’s integrity and the accuracy of the data analysis. No artificial intelligence was utilized in any aspect of this research. The opinions, results, and conclusions reported are those of the authors. No endorsement by ICES or its funders or data providers is intended or should be inferred. The authors declare that there are no personal or professional conflicts of interest regarding any aspect of this study. Complete disclosure of interest forms according to ICMJE are available on the article page, doi: 10.2340/17453674.2024.41506

## Results

**Figure 1:**
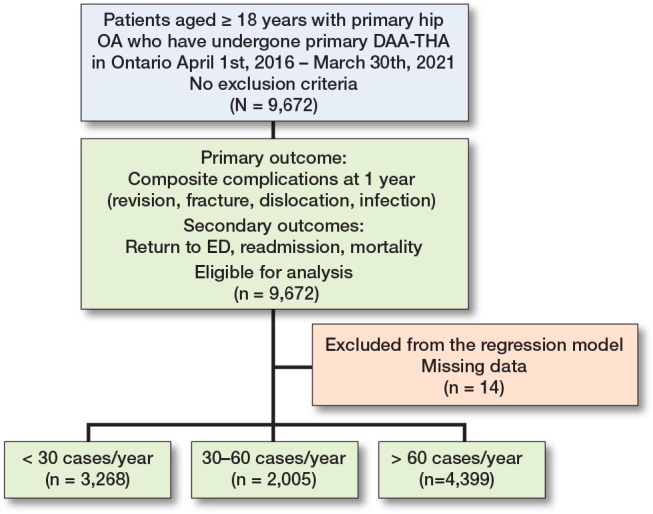
Flow diagram of this population-based retrospective cohort study. OA = osteoarthritis; DAA-THA = direct anterior approach total hip arthroplasty; ED = emergency department.

9,672 DAA-THA patients were included in the analysis, categorized by annual surgeon volume into < 30, 30–60, and > 60 cases/year (Figure 1). The median age of the participants was 67 years, with an interquartile range (IQR) of 59–74. Most of the cohort (80%) had a CCI of 0, and 43% of DAA-THA were performed in teaching hospitals (Table 1). The distribution of comorbid conditions and socioeconomic status showed slight variations across groups, without significant disparities. The number of DAA-THA performed annually markedly increased over the years, with a peak in 2019 followed by a drop in 2020, likely due to the coronavirus 2019 (COVID-19) pandemic.

**Table 1:** Preoperative characteristics of DAA-THA by surgeon volume group. Values are count (%) unless otherwise specified

Variables at index surgery	Overall N = 9,672	Surgeon DAA-THA volume, cases/year		
		< 30 n = 3,268	30–60 n = 2,005	> 60 n = 4,399
Age, median	67	68	68	66
IQR	59–74	60–75	60–74	58–73
Female	5,108 (53)	1,801 (55)	1,075 (54)	2,232 (51)
BMI > 40	519 (5.4)	216 (6.6)	106 (5.3)	197 (4.5)
CCI				
0	7,773 (80)	2,575 (79)	1,606 (80)	3,592 (82)
1	997 (10)	356 (11)	213 (11)	428 (9.7)
2	612 (6.3)	221 (6.8)	134 (6.7)	257 (5.8)
3	150 (1.6)	55 (1.7)	25 (1.2)	70 (1.6)
4	54 (0.6)	30 (0.9)	8 (0.4)	16 (0.4)
≥ 5	86 (0.9)	31 (0.9)	19 (0.9)	36 (0.8)
Diabetes	217 (2.2)	83 (2.5)	46 (2.3)	88 (2.0)
Hypertension	247 (2.6)	91 (2.8)	54 (2.7)	102 (2.3)
Rheumatoid				
arthritis	37 (0.4)	16 (0.5)	9 (0.4)	12 (0.3)
COPD	223 (2.3)	90 (2.8)	44 (2.2)	89 (2.0)
CVD	96 (1.0)	37 (1.1)	11 (0.5)	48 (1.1)
CHF	121 (1.3)	59 (1.8)	17 (0.8)	45 (1.0)
Connective tissue				
disease	84 (0.9)	27 (0.8)	22 (1.1)	35 (0.8)
Frailty	499 (5.2)	211 (6.5)	93 (4.6)	195 (4.4)
Year DAA-THA performed				
2016	1,285 (13)	753 (23)	225 (11)	307 (7.0)
2017	1,744 (18)	647 (20)	366 (18)	731 (17)
2018	2,396 (25)	693 (21)	526 (26)	1,177 (27)
2019	3,061 (32)	786 (24)	595 (30)	1,680 (38)
2020	1,186 (12)	389 (12)	293 (15)	504 (12)
Neighborhood income quintile				
1	1,289 (13)	441 (14)	282 (14.)	566 (13)
2	1,709 (18)	582 (18)	351 (18)	776 (18)
3	1,887 (20)	658 (20)	391 (20)	838 (19)
4	1,954 (20)	650 (20)	419 (21)	885 (20)
5	2,819 (29)	933 (29)	557 (28)	1,329 (30)
Index procedure performed in teaching				
hospitals	4,115 (43)	1,114 (34)	774 (39)	2,227 (51)

DAA-THA = direct anterior approach total hip arthroplasty;

IQR = interquartile range; BMI = body mass index; CCI = Charlson Comorbidity Index ;COPD = chronic obstructive pulmonary disease; CVD = cerebrovascular disease; CHF = congestive heart failure.

The influence of hospital teaching status on surgical outcomes reveals no significant difference in the rate of complications between teaching (2.4%) and non-teaching (2.6%) hospitals (CI –0.43% to 0.83%, P = 0.5). The overall complication rates within the first year after surgery were 3.1% (CI 2.5–3.7), 2.2% (CI 1.6–3.0), and 2.2% (CI 1.8–2.7) for the surgical experience group of < 30, 30–60, and > 60 cases/year, respectively (Table 2). Between-group pairwise differences in complication rates were 0.85% (CI –0.97 to 2.67) for < 30 vs 30–60 cases/year, 0.91% (CI –0.59 to 2.41) for < 30 vs > 60 cases/year, and 0.06% (CI –1.55 to 1.67) for 30–60 vs > 60 cases/year. Although the composite complication rate was low across all groups, the < 30 cases/year group had the highest incidence. The likelihood of visiting the ED within 30 days post-surgery was also higher in the lowest volume group. Length of stay in acute care and rehabilitation also varied with surgeon volume, with shorter stays observed in the higher volume groups.

**Table 2:** Postoperative secondary outcomes of DAA-THA by surgeon volume group

Secondary outcomes	Overall N = 9,672	Surgeon DAA-THA volume, cases/year		
		< 30 n = 3,268	30–60 n = 2,005	> 60 n = 4,399
Categorical outcomes, n (%)				
Individual surgical complication within 1 year				
Composite of complications	242 (2.5)	101 (3.1)	45 (2.2)	96 (2.2)
Deep infection	75 (0.8)	41 (1.3)	10 (0.5)	24 (0.5)
Dislocation	34 (0.4)	13 (0.4)	6 (0.3)	15 (0.3)
Hip fracture	42 (0.4)	14 (0.4)	8 (0.4)	20 (0.5)
Within 30 days of surgery				
Return to the ED	1,334 (13.8)	516 (16)	250 (13)	568 (13)
Readmission	382 (3.9)	159 (4.9)	73 (3.6)	150 (3.4)
Death within 1 year	67 (0.7)	25 (0.8)	14 (0.7)	28 (0.6)
Continuous outcomes, median (IQR), days				
Acute care LOS	1.59 (1.58)	2.03 (1.95)	1.65 (1.48)	1.23 (1.18)
Rehab. LOS	0.44 (3.60)	0.69 (4.34)	0.45 (3.39)	0.25 (3.03)

DAA-THA = direct anterior approach total hip arthroplasty; ED = emergency department; LOS = length of stay, IQR = interquartile range.

**Figure 2:**
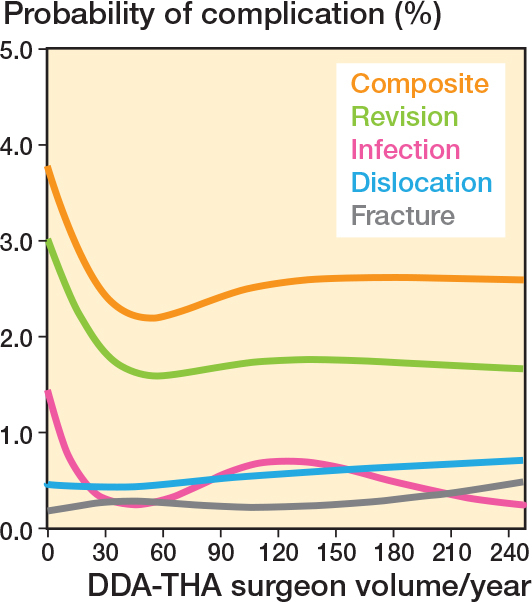
Composite and specific complications related to the direct anterior approach based on surgeon annual volume.

Analysis of the relationship between DAA-THA surgeon volume (cases/year) and the probability of major complications post-DAA-THA shows that as the surgeon volume increases, the probability of complications generally decreases at lower surgeon volumes of 0–30 cases/year (Figure 2). The trend persists for revision and infection, but not for dislocation and fracture. After the surgeon volume surpasses 30 cases/year, the probability of the composite complications slightly increases at 60 cases/year and reaches a plateau at the volume of approximately 120 cases/year. The composite line (top line in Figure 2) also showcases an association between surgeon volume and the likelihood of major complications after DAA-THA. It shows a distinct trend in the probability of complications as the surgeon volume changes. DAA-THA generally demonstrates a steep decline in complication probabilities in the lower range of surgeon volume, from 0 to 30 cases/year, where its nadir lies. After reaching the nadir, the probabilities of complications slightly increase. The rate of increase was relatively steeper at surgical volume between 60 and 120 cases/year and then reached a plateau at the surgical volume of 120 cases/year.

Both crude and adjusted ORs, using GEE to adjust for multiple comparisons and potential confounders, are shown in Table 3. The adjusted ORs for complications were 0.99 (CI 0.98–1.00; P = 0.2) for 30–60 cases/year and 0.99 (CI 0.98–1.00; P = 0.2) for > 60 cases/year, compared with the reference group of < 30 cases/year after adjusting for other baseline variables. No significant difference was found in adjusted complication rates between the groups.

**Table 3:** Crude and adjusted odds ratios (OR) of complications by surgeon volume categories and multivariable analysis of risk factors following DAA-THA using generalized estimating equations

Variable	OR (CI)	P value
**Crude OR (CI)**		
Surgeon volume, cases/year		
30–60	0.72 (0.50–1.03)	0.07
> 60	0.70 (0.53–0.93)	0.01
< 30	1 (Ref.)	
**Adjusted OR (CI)** ^a^		
Surgeon volume, cases/year		
> 60	0.99 (0.98–1.00)	0.2
> 60	0.99 (0.98–1.00)	0.2
< 30	1 (Ref.)	
Age	1.00 (1.00–1.00)	0.2
Sex		
Male	1.00 (0.99–1.00)	
Female	1 (Ref.)	
Charlson Comorbidity Index		
1	1.00 (0.99–1.01)	0.5
2	1.01 (0.99–1.03)	0.2
3	1.02 (0.97–1.06)	0.5
4	1.00 (0.96–1.04)	0.9
≥ 5	1.08 (0.97–1.21)	0.2
0	1 (Ref.)	
Comorbidities		
CHF	1.03 (0.98–1.09)	0.3
Diabetes	1.03 (0.99–1.08)	0.2
Obese	1.03 (1.00–1.05)	0.06
Frailty	1.01 (0.99–1.04)	0.2
Neighborhood income quintile		
1	0.99 (0.98–1.01)	0.3
2	0.99 (0.98–1.00)	0.2
3	1.00 (0.99–1.01)	0.8
4	1.00 (0.99–1.01)	0.5
5	1 (Ref.)	

OR = odds ratio; CI = 95% confidence interval.

a Adjusted estimates derived using least squares means to control for potential confounders.

## Discussion

We aimed to assess the association between the surgeon’s experience, represented by the annual surgical volume for DAA-THA, and the occurrence of postoperative major complications.

We found a significant impact of annual surgical volume on the incidence of major complications in DAA-THA. The risk for complications was highest for surgeons with relatively low annual volumes (< 30 DAA cases/year), with decreases in this risk with higher volumes. While the differences between volume categories were not significantly different in the adjusted analysis, this is likely due to lack of power. Overall, our observations are consistent with findings by Ravi et al., who highlighted a threshold for surgical volume in THA of mixed approaches at 35 cases per year, above which there is a noted reduction in dislocation and revision rates [10]. Klag et al. also reported a similar correlation, with fewer intraoperative femoral fractures associated with higher annual THA volumes [16]. The same direction of results between these previous studies and ours emphasizes the crucial role that the surgeon volume has across different THA approaches in determining patient outcomes. Regardless, it is important to note that the details of complications under our study were different than the others; while infections and early revisions contributed largely to our composite primary outcome, Ravi et al. and Klag et al. focused more on fractures and dislocations.

Our findings further highlight that a consistent surgical volume is one of the keys to mastering DAA-THA, as evidenced by the reduction in major complications observed when the annual case volume reaches and is maintained at 30 cases/year. Ongoing practice and maintaining adequate experience is indispensable for enhancing skills and minimizing complications, a notion not unique to DAA-THA but also recognized in other high-technical demanding lower limb reconstruction procedures, including hip resurfacing [17], Oxford mobile bearing unicompartmental knee arthroplasty [18-20], Sugioka’s femoral head rotational osteotomy for osteonecrosis [21], and other complex hip reconstructive surgeries [22, 23].

Interestingly, after the surgical volume exceeds 60 cases/year or the nadir, we found an increase in the probability of complications following DAA-THA. This resurgence in complications might have come from the surgeons’ growing confidence to undertake more challenging and difficult cases after surpassing the initial learning curve. The main underlying issue lies in appropriate patient selection for DAA-THA. Ideal candidates for DAA-THA likely include OA hip patients with relatively thin subcutaneous fat, valgus and long femoral neck, and no pelvic overhang. Conversely, patients with obesity, varus femoral neck, shorter femoral neck, and pelvic overhang may present increased challenges for DAA-THA, possibly leading to higher complication rates.

The observed pattern is consistent with trends seen in several surgical approaches, indicating that the rise in complication rates is probably due to broader patient selection criteria and inherent difficulties of more complicated patients, rather than being caused by the DAA technique itself. This pattern aligns with trends observed across various surgical approaches by the study from Ravi et al., suggesting that the increase in complication rates is likely a function of expanded patient selection criteria and the inherent challenges of more complex cases [10]. Similarly, as surgeons gain proficiency and confidence, they might attempt DAA-THA on those with less-than-ideal anatomical features for the procedure that inherently carry higher risks.

While our findings align with several studies, there exist studies with other interesting findings that we did not explore. Markel et al., for instance, discussed the superior outcomes among centers with “surgeon champions,” defined as surgeons who play an influential role as liaisons and advocates within the arthroplasty registry sites, emphasizing that the participation in quality improvement initiatives can potentially enhance surgeons’ performance and improve patient outcomes [24]. However, we did not specifically investigate this concept. We reviewed data obtained from a central database in Ontario without this variable, thus not being able to explore this potential association.

Moreover, we also found discrepancies in the threshold of surgical volume to minimize complications post-DAA-THA compared with others. De Steiger et al. reported a learning curve for the anterior THA approach, suggesting that at least 50 procedures are required to be performed by a surgeon before their revision rate matches that of the surgeon having performed 100 or more procedures [7]. Similarly, a systematic review by Nairn et al. demonstrated a substantial learning curve associated with DAA-THA at approximately 100 cases, after which the operative time reached a plateau and complication rates decreased significantly [8]. However, in the present study, we observed that the surgical volume needed to achieve the learning curve for DAA-THA was approximately at least 30 cases/year, notably less than the 100 suggested by previous studies. One potential explanation for this discrepancy could have been the advancement of learning platforms and modalities. With a lot of attention on DAA-THA in recent years, there has been an increasing trend towards this surgical technique employed worldwide. Therefore, surgeons could have had more accessible avenues to advance their skills and understand the key points of this procedure. The availability of tips, tricks, and best practices online might also have shortened the learning curve, as surgical techniques are disseminated more widely and efficiently than in the past. On top of that, recent and current trainees are more likely to be exposed to DAA-THA early in their training, which may result in faster ascent to competence. For these reasons, we might be expecting an even faster learning curve in the near future.

### Strengths

The main strength of our study was that we established the learning curve of DAA-THA using province-wide and diverse population-based data involving surgeons of various expertise. It should be emphasized that recognizing the distinct learning curves associated with each THA approach is crucial. Although a previous population-based study has already reported the relationship between successful THA and surgical volume, it utilized a cohort of mixed THA approaches without evaluating the learning curves specific to each type of THA [10]. Many other previous studies have already evaluated the learning curve associated with the DAA technique based on individual surgeons or a limited number of surgical centers [25-28]. However, with our population-based dataset, we are able to capture a wider variety of surgical scenarios, outcomes, and patient demographics. We are also able to capture complications that presented and were managed at centers different from where the primary procedure was performed. Therefore, we are among the first to report this association specific to DAA-THA using a population-based sample, thus providing robust estimates that offer more focused and relevant implications for those considering using this technique.

### Limitations

First, our analytical approach using restricted cubic splines, though accommodating potential non-linear relationships, might be perceived as more intricate and less intuitive compared with the traditional linear regression. Second, the analyses by surgeon volume categories led to smaller sample size, thereby limiting the study power and potentially producing less reliable confidence intervals and P values. Therefore, the interpretation of odds ratio with 95% CI results should be approached cautiously due to the limited sample size for specific subgroups. Third, our study may not have captured all complications related to DAA-THA. Specifically, conditions important to patients despite not requiring surgical interventions, such as numbness due to lateral femoral cutaneous nerve irritation, were not recorded. This oversight exemplifies a common challenge in retrospective studies, which may not always capture less apparent but clinically relevant complications. Fourth, our study primarily pertains to primary DAA-THA procedures, thus restricting its generalizability to other THA approaches or THA integrated with other techniques, such as computer or robotic-assisted THA or revision surgeries. Fifth, our dataset categorizes the surgical approach based on Ontario’s data registry, which could not distinguish between variations in the skin incision, such as the standard versus bikini incision. While our study focused on the direct anterior approach, we also lacked comparative data on DAA usage relative to other surgical techniques among participating hospitals. Future studies could benefit from examining this aspect to deepen our understanding of different THA approaches and outcomes. Lastly, in our analysis, “revision arthroplasty” is defined broadly to include all causes of surgical revision. While our study captures major complications within the first year post-surgery, it is important to note that certain complications, such as aseptic loosening, typically develop over a longer period and may not be fully observable within our 1-year follow-up timeframe.

### Conclusion

There was an inverse relationship between surgical volume and complication rates in DAA-THA within the lower volume ranges.

In perspective, this extensive population-based study underscores the significant impact of surgical volume on major complications following DAA-THA. Maintaining a surgical volume of at least 30 DAA-THA cases per year can minimize complications, emphasizing the importance of surgical volume in this approach.

## Notes

PR: conceptualization, investigation, methodology, validation, visualization, original writing, editing and rebuttal, data curation, and project administration. EB: validation, preliminary draft and results report. DP: data curation, project administration, supervision. MP: data curation, validation, data curation, and formal analysis. BR: conceptualization, data curation, formal analysis, investigation, methodology, project administration, resources, supervision, validation, editing and rebuttal.

The authors are grateful for Amreen Babujee’s dedication and generous support in this endeavor. Parts of the material are based on data and information compiled and provided by the Canadian Institute for Health Information (CIHI). This study was supported by ICES, which is funded by an annual grant from the Ontario Ministry of Health and the Ministry of Long-Term Care. Parts of this material are based on data and/or information compiled and provided by the Ontario Ministry of Health and the Canadian Institute for Health Information. This document used data adapted from the Statistics Canada Postal Code Conversion File, which is based on data licensed from Canada Post Corporation, and/or data adapted from the Ontario Ministry of Health Postal Code Conversion File, which contains data copied under license from Canada Post Corporation and Statistics Canada. The analyses, conclusions, opinions, and statements expressed herein are solely those of the authors and do not reflect those of the funding or data sources; no endorsement is intended or should be inferred.

Handling co-editors: Keijo Mäkelä and Robin Christensen

*Acta* thanks Mona Badawy and Martin Buttaro for help with peer review of this manuscript.
